# Evaluation of Virus Inactivation by Formaldehyde to Enhance Biosafety of Diagnostic Electron Microscopy

**DOI:** 10.3390/v7020666

**Published:** 2015-02-10

**Authors:** Lars Möller, Livia Schünadel, Andreas Nitsche, Ingeborg Schwebke, Manuela Hanisch, Michael Laue

**Affiliations:** 1 Advanced Light and Electron Microscopy (ZBS 4), Robert Koch Institute, Berlin D-13353, Germany; E-Mail: LaueM@rki.de; 2 Highly Pathogenic Viruses (ZBS 1), Robert Koch Institute, Berlin D-13353, Germany; E-Mails: SchuenadelL@rki.de (L.S.); NitscheA@rki.de (A.N.); 3 Hospital Hygiene, Infection Prevention and Control (FG 14), Robert Koch Institute, Berlin D-13353, Germany; E-Mails: SchwebkeI@rki.de (I.S.); HanischM@rki.de (M.H.)

**Keywords:** formaldehyde, inactivation, plaque assay, TCID50 assay, diagnostic electron microscopy, negative staining, biosafety, virus

## Abstract

Formaldehyde (FA) fixation of infectious samples is a well-established protocol in diagnostic electron microscopy of viruses. However, published experimental data that demonstrate virus inactivation by these fixation procedures are lacking. Usually, fixation is performed immediately before the sample preparation for microscopy. The fixation procedure should transform viruses in a non–infectious but nonetheless structurally intact form in order to allow a proper diagnosis based on morphology. FA provides an essential advantage in comparison to other disinfectants, because it preserves the ultrastructure of biological material without interfering significantly with the preparation (*i.e*., the negative staining) and the detection of viruses. To examine the efficiency of FA inactivation, we used *Vaccinia virus*, *Human* *adenovirus* and *Murine norovirus* as models and treated them with FA under various conditions. Critical parameters for the inactivation efficiency were the temperature, the duration of the FA treatment, and the resistance of the virus in question. Our results show that FA inactivation at low temperature (4 °C) bears a high risk of incomplete inactivation. Higher temperatures (25 °C) are more efficient, although they still require rather long incubation times to fully inactivate a complex and highly robust virus like *Vaccinia*. A protocol, which applied 2% buffered FA for 60 min and a temperature–shift from 25 to 37 °C after 30 min was efficient for the complete inactivation of all test viruses, and therefore has the potential to improve both biosafety and speed of diagnostic electron microscopy.

## 1. Introduction

Diagnostic electron microscopy of viruses is used as a “scouting” method in situations or diseases where speed of diagnostics is critical (e.g., in outbreaks of highly contagious diseases), or where the diagnosis remains unclear even after a first analysis by alternative screening techniques [1,2,3,4]. The key method in diagnostic electron microscopy is negative staining, and this mostly because the method is robust and well established [5,6,7], with extensive reference data available [8,9,10]. The main advantages of diagnostic negative staining electron microscopy are processing time (a single sample can be prepared and analyzed within an hour) and its generic approach, which means that electron microscopy (EM) is potentially able to detect all virus particles present in a sample, even viruses that are unknown or unsuspected [2,4]. Identification is based on morphological criteria allowing assignment of viruses to a morphological group, which is usually identical with the taxonomical category of a virus family [8]. Although this procedure does not allow identification of a specific virus species or strain, the diagnosis of the virus family (e.g., poxvirus* versus* herpesvirus) already helps to expedite a more specific diagnosis by providing an evidence–based hypothesis, which, for instance, facilitate the selection of reagents (such as those for PCR and immunological tests) for more specific identification down to the virus genus or species.

Exposure of lab personnel and the public by accidentally released viruses during handling of virus–infected biological material must be avoided by efficient and feasible safety precautions. Since most electron microscopes are operated at biosafety level two and below, proper sample inactivation is necessary to guarantee biosafety [11,12,13]. For diagnostic purposes, the inactivation procedures must not only inactivate the viruses, but simultaneously preserve their morphology. Aldehyde fixation is well established in histology and EM [14]. In diagnostic negative staining EM, FA at a concentration of 2% to 4% is used because it stabilizes biological structures [14], and its virucidal efficiency is very well documented, e.g., in vaccination programs [15,16,17,18,19]. Glutaraldehyde (GA) is another very efficient inactivating agent if applied at a concentration of a few volume percent [20,21,22], but likely to cause aggregates and masks virus structures that are crucial for diagnosis [3,23]. Other inactivating procedures, like the application of heat, alcohol, peroxide, or radiation may impair the structural features of the viruses [20] and are, therefore, not useful for diagnostic EM. Published data on the kinetics and concentration dependency of FA inactivation [24] suggest that the inactivation by classical FA–fixation may be effective. However, an experimental proof for the inactivation efficiency of the FA–fixation protocols used in diagnostic EM is still lacking.

We therefore started to evaluate the inactivation efficiency of FA–fixation by using three different model viruses known for their environmental stability: a complex enveloped virus (*Vaccinia virus*), a standard test virus in disinfection studies (*Human adenovirus*) and a more robust non–enveloped virus (*Murine norovirus*). Virus infectivity was determined before and after treatments by standardized methods (plaque or TCID50 assay). Structural integrity of inactivated viruses was checked by negative staining EM. As a main result of our study, we provide a rapid (1 h) inactivation protocol using 2% buffered FA, which completely inactivates concentrated virus suspensions.

## 2. Experimental Section

### 2.1. Virus Stocks and Cell Culture Systems

Three different viruses were used for testing the inactivation efficiency. One complex, enveloped virus, *Vaccinia virus* (New York City Department of Health Laboratories; ATTC VR–1536), and two non–enveloped viruses, *Murine norovirus* (S99; Berlin/2006/DE) and *Human adenovirus* type 5 (Adenoid 75 strain; ATCC VR–5). Three different poxvirus strains were used as additional positive control in each plaque assay: *Cowpox virus* (EP–GuWi; CPXV EleGri07/1 [25]), *Cowpox virus* (Brighton; ATCC VR–302) or *Vaccinia virus* (Lister Elstree; Bavarian*-*Nordic). Poxviruses were propagated in Hep2 cells (HeLa derivative; ECACC, No. 86030501), and plaque assays were performed using Vero C1008 cells (ECACC, No. 85020206). *Murine norovirus* was proliferated in RAW 264.7 cells (ATCC TIB–71) and *Human adenovirus* in A549 cells (ATCC CCL–185). Tissue culture infective dose 50 (TCID50) assays were conducted using the same cells, respectively.

### 2.2. Virus Propagation and Harvesting

#### 2.2.1. Vaccinia Virus

Hep2 cells infected with *Vaccinia virus* (MOI 0.5) were cultivated in Dulbecco’s Modified Eagle Medium (D–MEM), including 1% L-glutamine, (Gibco/Invitrogen) and 5% fetal bovine serum (PAA) for four days at 37 °C and 5% CO_2_. Cells with media of several culture flasks (175 cm^2^) were frozen/thawed thrice, scraped and pooled. Cell debris was sedimented by low speed centrifugation at 724 *g* for 10 min, and the supernatant was ultracentrifuged at 20,000 rpm for 1.5 h (pre-cooled SW 32 Ti rotor, Beckman Coulter) at 4 °C. The pellets were resuspended in D–MEM and stored at −80 °C until use as the stock solution for inactivation experiments. Virus titers were determined by plaque assay and ranged between 1.2 × 10^8^ and 1.3 × 10^9^ plaque forming units (PFU) per milliliter.

#### 2.2.2. Human Adenovirus

A549 cells were infected with *Human adenovirus* type 5 and maintained in D–MEM including 1% L-glutamine, 1% non–essential amino acid cell culture supplement (100 × concentrate; PAA), 0.11% NaHCO_3_ and 2% fetal calf serum for 3–5 days at 37 °C and 5% CO_2_. Cells were scraped and centrifuged at 4696 g for 15 min. The pellets were frozen/thawed thrice and centrifuged again at 4696 g for 15 min. Supernatant was frozen at −80 °C and stored until usage as the stock solution for inactivation experiments. The virus titer was determined by tissue culture infective dose 50 (TCID50) assay and ranged between 1 × 10^9^ and 1 × 10^10^ TCID50/mL.

#### 2.2.3. Murine Norovirus

*Murine norovirus* was propagated in Raw 264.7 cells, which were cultivated in Minimum Essential Medium (MEM) with Hank’s balanced salts (Invitrogen, 11012-044) and HEPES buffer, including 1% L-glutamine (200 mM; PAA), 1% sodium pyruvate (100 mM; PAA), 1% non–essential amino acid cell culture supplement (100×; PAA), 0.15% NaHCO_3_ and 3% fetal calf serum (Premium South Africa low endotoxin; PAN Biotech) for 3–5 days at 37 °C and 5% CO_2_. Cells were scraped and centrifuged at 4696 *g* for 15 min. The pellets were frozen/thawed thrice and centrifuged again at 4696 *g* for 15 min. Supernatant was stored at −80 °C until usage as the stock solution for inactivation experiments. The virus titer of the virus preparations ranged between 1 × 10^7^ and 1 × 10^8^ TCID50/mL.

### 2.3. Inactivation of Virus Suspensions

Formaldehyde inactivation solution was prepared from a frozen stock solution of 20% paraformaldehyde (PFA; Roth, No. 0335.3) in 0.5 M HEPES buffer (pH 7.2), which was heated to 60–70 °C for 20 min to shift the equilibrium towards non-polymerized FA. In the case of *Vaccinia virus,* a defined volume of the FA stock solution was added to samples (*i.e.*, 1.8 mL for tests shown in Table 1 or 0.9 mL in Table 4) of the virus stock solution and thereby diluted to the desired end concentration (*i.e.*, 2% or 4% FA in 0.05 M HEPES buffer). Immediately after the end of incubation time (for inactivation conditions, refer to Table 1,Table 2,Table 3 and Table 4) the test suspension was sedimented by ultracentrifugation at 65,000 rpm for 45 min at 4 °C (pre–cooled TLA–120.2 rotor, Beckman Coulter), collected in fresh D–MEM and frozen at −80 °C to quickly slow down chemical reactions and to prevent molecular mobility, which avoids reaction of free FA molecules with viruses. To estimate possible loss of infectious virions caused by ultracentrifugation, samples (2 and 1 mL) from one virus stock solution were ultracentrifuged, recovered in medium and analyzed by plaque assay. Infectivity of the two centrifuged samples was the same as infectivity of the virus stock solution (*i.e.*, 2 × 10^8^ PFU/mL). In the case of *Human adenovirus* and *Murine norovirus*, the PFA stock solution was diluted in HEPES buffer to a concentration of 2.2%, and nine units of the diluted PFA were mixed with one unit of the virus stock solution according to the DVV/RKI Guideline [26].

**Table 1:** Efficiency of *Vaccinia virus* inactivation by 2% formaldehyde (if not stated otherwise) in relation to temperature and incubation time. Virus titer was determined by plaque assays.

Temperature (°C)	Incubation Time (h)	Virus Titer (PFU/mL)	Virus Titer after Inactivation (PFU/mL)	Reduction at log_10_ Scale
4	2	1.2 × 10^8^	1.3 × 10^3^	4.9
4	2	1.2 × 10^8^	1.8 × 10^3^	4.7
4	48	1.2 × 10^8^	2.6 × 10^3^	4.5
4	48	1.2 × 10^8^	2.1 × 10^3^	4.6
4	168	1.2 × 10^8^	1.4 × 10^3^	4.9
4	168	1.2 × 10^8^	9.8 × 10^2^	5.2
25	2	1.6 × 10^8^	no plaques	complete
25	2	1.6 × 10^8^	no plaques	complete
25 (*)	3	1.3 × 10^9^	1.1 × 10^1^	8.2
25 (*)	3	1.3 × 10^9^	no plaques	complete
25	4	1.4 × 10^8^	2.5 × 10^2^ (***)	5.6
25	4	1.4 × 10^8^	no plaques	complete
25	4	1.4 × 10^8^	no plaques	complete
25	4	1.4 × 10^8^	no plaques	complete
25	4	1.6 × 10^8^	no plaques	complete
25	4	1.6 × 10^8^	no plaques	complete
25	6	1.4 × 10^8^	2.5 × 10^2^ (***)	5.6
25	6	1.4 × 10^8^	no plaques	complete
25	6	1.4 × 10^8^	no plaques	complete
25	6	1.4 × 10^8^	no plaques	complete
25	6	1.3 × 10^9^	no plaques	complete
25	6	1.3 × 10^9^	no plaques	complete
25	24	1.3 × 10^9^	no plaques	complete
25	24	1.3 × 10^9^	no plaques	complete
25/37	0.5/0.5	1.6 × 10^8^	no plaques	complete
25/37	0.5/0.5	1.6 × 10^8^	no plaques	complete
25/60	1/2	1.3 × 10^9^	no plaques	complete
25/60	1/2	1.3 × 10^9^	no plaques	complete
37	0.5	1.4 × 10^8^	no plaques	complete
37	0.5	1.4 × 10^8^	no plaques	complete
37	1	1.6 × 10^8^	no plaques	complete
37	1	1.6 × 10^8^	no plaques	complete
37 (**)	0.5	1.4 × 10^8^	1.5 × 10^3^ (***)	4.9
37 (**)	0.5	1.4 × 10^8^	no plaques	complete
37 (**)	0.5	1.4 × 10^8^	no plaques	complete
37 (**)	0.5	1.4 × 10^8^	no plaques	complete

(*) 4% FA; (**) 2% FA + 0.05% GA; (***) Note that residual infectivity was only detected at higher dilutions of the tests suspension in the plaque assay.

**Table 2:** Efficiency of *Human adenovirus* inactivation by formaldehyde at different temperatures. Virus titer was determined by TCID50 assay.

Treatment	Temperature (°C)	Incubation Time (h)	Virus Titer (log_10_ TCID50/mL)
-	4	1	8.9
2% FA	4	1	no CPE
2% FA	4	1	no CPE
-	25	1	8.5
2% FA	25	1	no CPE
2% FA	25	1	no CPE
-	25/37	0.5/0.5	8.9
2% FA	25/37	0.5/0.5	no CPE
2% FA	25/37	0.5/0.5	no CPE

CPE = cytopathic effect; Neutralization and toxicity tests performed as expected.

**Table 3:** Efficiency of *Murine norovirus* inactivation by formaldehyde at different temperatures. Virus titer was determined by TCID50 assay.

Treatment	Temperature (°C)	Incubation Time (h)	Virus Titer (log_10_ TCID50/mL)
-	4	1	7.2
2% FA	4	1	4.4
2% FA	4	1	4
-	25	1	7.6
2% FA	25	1	no CPE
2% FA	25	1	no CPE
-	25/37	0.5/0.5	7.4
2% FA	25/37	0.5/0.5	no CPE
2% FA	25/37	0.5/0.5	no CPE

CPE = cytopathic effect; Neutralization and toxicity tests performed as expected.

**Table 4:** Reproducibility of formaldehyde (2%) inactivation of *Vaccinia virus*. Virus titer was determined by plaque assay. Virus titer prior inactivation was 2.1 × 10^8^ PFU/mL.

Temperature (°C)	Incubation Time (h)	Number of Tests Showing Plaques after Treatment (*n* = 10)
4	1	1
25°	24	0
25/37°	0.5/0.5	0

All controls showed expected values.

### 2.4. Determination of Virus Infectivity

#### 2.4.1. Plaque Assays of Vaccinia Virus Infected Vero Cells

Plaque assays were performed according to [27]. Prior to inoculation all samples were ultrasonicated (Branson Sonifier 450) until they were homogenized,* i.e.*, no visible aggregates could be detected in the suspensions. For every FA-treated test suspension a 24–well plate (Nunc, Thermo Scientific) was prepared with 200 µL per well of a Vero cell suspension (1.2–1.5 × 10^6^ cells/mL) supplemented with 5% streptomycin-penicillin (100× concentrate; PAA) followed by 200 µL of the virus suspension at three dilutions (1:10, 1:20, 1:200). The dilutions were generated in duplicates and distributed in four different wells (Figure S1 B–F). In our initial experiments we used a dilution of 1:2 but observed a cytotoxic effect most probably caused by residual FA molecules and therefore used dilutions equal to or above 1:10 in further experiments. Treated cells were incubated for four hours at 37 °C and 5% CO_2_ and then covered with 400 µL of 1.6% carboxymethyl cellulose solution in medium (BDH, No. 27649). After four days of incubation, cells were fixed with 3.7% formalin (Roth, P733.2) for 30 min, stained with amido black 10B (naphthalene black; Sigma, N3393-100G), washed with water, and plaques were counted manually. Untreated virus suspensions (virus stock solution of the test suspensions and control virus suspensions of established infectivity; see Section 2.1. for a detailed description of the viruses) were tested in the same manner in a 24–well plate by tenfold serial dilution 1:10^3^ to 1:10^8^ (Figure S1 A). Virus titers were calculated as plaque forming units per volume (PFU/mL), whereas either 1:10 or 1:20 and 1:200 dilutions were considered in case of the inactivated test suspensions. The titer reduction (in log_10_ scale) was calculated relative to the virus titer estimated for the untreated virus suspension [28]. To increase statistical validity, selected inactivation tests were reproduced in ten separate assays each. In addition, a slightly modified plaque assay with an increased number of independent dilutions (four instead of two) of the test suspensions was applied (Table 4). In those assays virus was diluted 1:10 and 1:100 for test suspensions inactivated at 4 °C. For samples inactivated at 25 or 37 °C the 1:100 dilution was not done, because negative results were expected.

#### 2.4.2. Determination of Tissue Culture Infective Dose 50 (TCID50) for Murine Norovirus and Human Adenovirus

Tissue culture infective dose 50 (TCID50) was performed according to [26,29]. Cells were seeded in 96–well plates (12 rows × 8 columns; TPP Lot 92096) and cultivated for one day at 37 °C and 5% CO_2_. Rows 9 to 11 were left blank to avoid contamination of the negative control (100 µL of virus-free medium), which was placed in row 12. The test suspension was sequentially diluted tenfold (from 1:10 to 1:10^8^) in medium (900 µL + 100 µL test suspension), supplemented with 0.5% (w/v) L-histidine to neutralize free aldehyde and 2% (*Human adenovirus*) or 3% (*Murine norovirus*) fetal calf serum. All dilutions were tested eightfold in a single 96–well plate. Untreated virus suspensions were tested in the same manner. Cytopathic effect (CPE) was evaluated after seven days for *Human adenovirus* and after five days in case of *Murine norovirus* using an inverted microscope. The virus titer was determined using the method of Spearman [30] and Kaerber [31] and expressed as tissue culture infective dose 50 per mL (TCID50/mL), which is the concentration that kills 50% of the cells. For the validation of the neutralization efficiency 8.9 mL L-histidine (0.5% w/v) + 1 mL of 2% PFA were incubated for 5 min at 20 °C. Then 100 µL of a 1:100 dilution of unfixed virus suspension were added and incubated for 30 min. Toxicity of the neutralizer was proven as follows: 9.9 mL L-histidine (0.5% w/v) + 100 µL of a 1:100 dilution of unfixed virus suspension were incubated for 30 min. 10–fold dilutions of these validation tests and the untreated virus suspension were titrated on the related cells, respectively, to prove the correctness of test performance.

### 2.5. Negative Staining Transmission Electron Microscopy

Negative staining transmission electron microscopy of the virus stock solutions and test suspensions was performed according to [5]. A drop (10 µL) of the test suspension was placed directly onto a glow-discharged EM sample support (400 mesh copper grid, covered with a carbon reinforced plastic film). After adsorption for 10 min at room temperature, the grids were washed three times in double distilled water and negatively stained with 2% uranyl acetate, pH 4.5 (1% in case of *Vaccinia virus*). The grids were examined using a JEM–2100 transmission electron microscope (JEOL Corp.) operated at 200 KV. Micrographs were recorded with a Veleta CCD camera (Olympus Soft Imaging Solutions) at a resolution of 2048 × 2048 pixels.

## 3. Results

In electron microscopy, fixation is frequently performed at room temperature or even at 4 °C [32]. However, only few data are available on the fixation efficiency at different temperatures, which do not allow general conclusions ([33], page 57). To get an idea about the temperature/time dependence of the inactivation efficiency of the preferred formaldehyde (FA) fixation used in negative staining EM, we treated *Vaccinia virus*, *Murine norovirus* and *Human* *adenovirus* suspensions with 2% buffered FA at 4 and 25 °C for different periods. The experiments demonstrated that short incubation times (one or two hours, respectively) at 4 °C are not sufficient to completely suppress infectivity of high concentrations (above 10^7^ PFU/mL) of *Murine norovirus* and *Vaccinia virus* (Table 1 and Table 3), while *Human* *adenovirus* is inactivated after a FA treatment at 4 °C for one hour (Table 2). At 25 °C some of the *Vaccinia virus* test suspensions showed residual infectivity, even if longer incubation times (up to six hours) were applied. Complete inactivation of *Vaccinia virus* infectivity was achieved by an incubation for 24 h at 25 °C (Table 1). Infectivity of *Human* *adenovirus* and *Murine norovirus* was not observed after FA treatment of suspensions for at least one hour at 25 °C (Table 2 and Table 3).

Diagnostic electron microscopy of viruses is mainly used in emergency situations and therefore needs to be performed as quickly as possible. To find efficient and short inactivation conditions for inactivation by FA, we tested a higher concentration of FA, a combination with low concentration of glutaraldehyde (0.05%) and/or temperatures above 25 °C with the most resistant virus of our test viruses,* i.e.*, *Vaccinia virus*. Our preliminary experiments suggested that temperature would be the most efficient parameter to accelerate a complete inactivation of highly concentrated *Vaccinia virus*. Incubations using 2% buffered FA at 37 or 60 °C for at least 30 min were sufficient to inactivate virus infectivity in a number of experiments (Table 1). Other strategies to increase the speed of inactivation failed. In the experiment, in which 0.05% GA was added to the 2% buffered FA, the virus suspension was not completely inactivated within 30 min at 37 °C, suggesting interference of the FA inactivation by GA or failure of the FA inactivation within the 30 min incubation time. Application of higher FA concentration,* i.e*., 4% FA, for 3 h at 25 °C was also not sufficient to block infectivity of the test suspension (Table 1). The efficiency of FA inactivation at higher temperature was also tested for *Murine norovirus* and *Human adenovirus*. No cytopathic effect in TCID50 assays could be detected after treatment of virus suspensions with 2% buffered FA for 30 min at 25 °C followed by 30 min at 37 °C (Table 2 and Table 3).

In order to ascertain the relevance of our findings, we repeated selected inactivation schemes in ten independent inactivation experiments, each using *Vaccinia virus* as a model and a slightly modified plaque assay (see Section 2.4.1. for details). Besides inactivation at 4 °C for one hour, tests at 25 °C for 24 h and a temperature–shift protocol (0.5 h for 25 and 0.5 h at 37 °C) were performed. Virus infectivity was found in one of ten samples inactivated at 4 °C. This residual activity was shown consistently in all of the four independent dilutions of the sample which were deployed for plaque testing. All of the other test suspensions did not show any residual infectivity (Table 4). Controls, especially controls using different untreated poxvirus suspensions (see Section 2.4.1. for details), showed the expected results, indicating no significant bias in the detection of virus infectivity.

**Figure 1:**
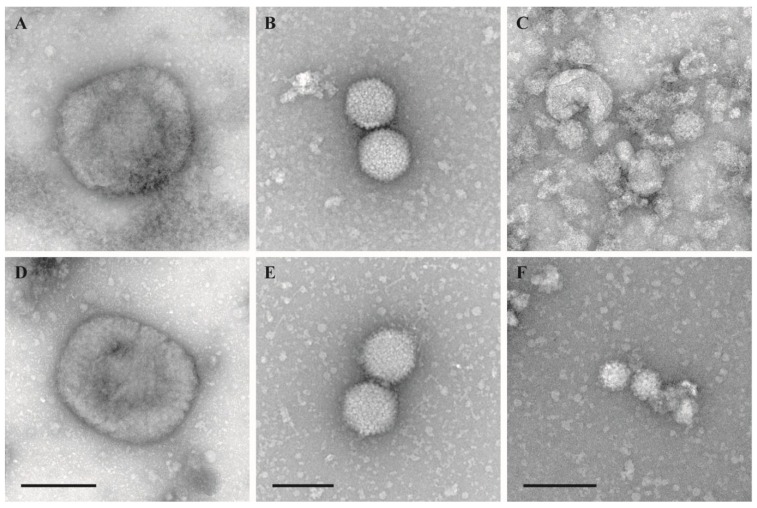
Negative staining EM of untreated and treated virions. (**A**–**C**): Untreated stock suspensions; (**D**–**F**): Test suspensions treated with 2% FA at 25/37 °C for 30 min at each temperature (temperature-shift protocol); (A) and (D): *Vaccinia virus* VR–1536, bar = 200 nm; (**B**) and (**E**): *Human adenovirus* type 5, adenoid 75 strain, bar = 100 nm; (**C**) and (**F**): *Murine norovirus* S99, bar = 100 nm.

The morphological integrity of virus particles was checked by negative staining electron microscopy. Virus morphology was not affected by the inactivation using the temperature–shift protocol (25/37 °C, 0.5/0.5 h) (Figure 1). Samples inactivated by other protocols were only occasionally evaluated by negative staining EM but never revealed any significant structural impairment.

## 4. Discussion

Our results show that the inactivation of virus suspensions by FA depends on temperature, incubation time and the type of virus. This result and the influence of other factors, like pH and FA concentration on inactivation efficiency, have been documented by other studies before (reviewed in [15,24,34]) and therefore was not surprising. However, we showed that a buffered FA solution of 2%, which is commonly applied in diagnostic electron microscopy of viruses, needs at least room temperature (*i.e*., 25 °C) and rather long incubation time (more than six hours) to completely inactivate complex viruses such as the *Vaccinia virus*. Although temperature dependency of FA inactivation is known, it came as a surprise that even an exposure of *Vaccinia virus* to 2% FA for several days at 4 °C did not fully inactivate the virus. The non–enveloped viruses tested were much more susceptible to FA treatment at low temperature than the enveloped *Vaccinia virus*, but showed also differences in their reactivity, indicating slow reactivity of the FA at 4 °C. In summary, we concluded that inactivation of viruses at 4 °C should be avoided. Inactivation at 25 °C was much more efficient but still needed several hours for a complete inactivation of concentrated suspensions of the complex *Vaccinia virus*. A significant reduction in incubation time to reach complete inactivation was found with a further increase of temperature to 37 °C after a pre-treatment at 25 °C (temperature–shift protocol). With a total inactivation time of 60 min, the temperature-shift protocol becomes useful for emergency diagnostics. It seems likely that further modifications of the protocol, like increasing temperature to higher levels than 37 °C, could shorten the complete inactivation even further. Increasing the FA concentration (e.g., to 10%) could also accelerate inactivation [35]. However, the rapidity of inactivation must be carefully balanced against the residual risk of incomplete inactivation, ultrastructural preservation and visibility, which is necessary for the generic detection capabilities of diagnostic EM.

It was not our goal to study the virucidal properties of FA in detail, but rather to develop an inactivation protocol that is useful in practice. The temperature-shift protocol provides appropriate speed and safety especially for application in emergencies. Inactivation at 25 °C may also be used for diagnostic samples if incubation time can be extended to 24 h. However, the following concerns remain and need further investigation:

(1) The inactivation of viruses by FA has been evaluated by using three different test viruses, and results showed that the inactivation efficiency is highly dependent on the virus type, as expected (e.g., [36]). While our results allow already generalized conclusions, tests using other viruses, even more resistant viruses than poxviruses (e.g., small non-enveloped viruses, like parvo- and circoviruses [34,37]), must be performed to prove the inactivation efficiency of the so far successful protocols.
(2) The inactivation tests have been conducted using non–purified cell culture supernatants. Although these samples contain biological macromolecules besides virus particles released by the cells in culture, concentration of such material is rather low in comparison to some diagnostic samples, e.g., serum or stool. Therefore, additional experiments, using defined loads of protein or other biological material (e.g., urine, cell or tissue homogenates), must be performed to determine the possible interference of the inactivation by high concentrations of other biological material.
(3) Concentration of viruses may also be a critical factor for inactivation efficiency. To produce high concentrations and sufficient amount of active test viruses is difficult and laborious. In our tests, we used concentrations up to 10^9^ PFU/mL of the highly stable poxviruses. Although the situation of diagnosing a sample with higher virus load seems rather unlikely, it should be investigated whether the proposed inactivation protocols are efficient at even higher concentrations or not.
(4) Our results showed a slight variability in the inactivation efficiency, even under ideal experimental conditions. The poxvirus suspension used for the reproduction experiments (Table 4) was more sensitive for FA inactivation at low temperature than the poxvirus suspension used in the preliminary experiments (Table 1). Variability may be due to differences in the sensitivity of varying virus batches, which has been suggested before [24]. Another possibility could be a difference in the homogenization efficiency of the different virus batches, which could result in a variable number and size of virus aggregates. Aggregation is a well-known factor, which affects inactivation efficiency of many disinfectants [38] and could be the reason for the observed residual infectivity in few of the treated *Vaccinia virus* suspensions which became apparent only at higher dilutions in the plaque test. Higher dilution could have promoted disintegration of aggregates and release of infectious particles. Further experiments must consider these variables and should provide an estimate regarding their effects.
(5) For an application of the suggested inactivation protocols in emergency diagnostics, the inactivation efficiency of the protocols must be tested also for bacteria, since a pre-screening of the samples for bacteria is not possible in any case. From the published data on FA inactivation, at least a significant reduction of vegetative bacteria can be expected [39,40]. Bacterial spores are much more resistant to FA inactivation than their vegetative forms (for a review see [41]), but FA is able to penetrate even the spore core, which houses the DNA [42]. Increased incubation temperature may help to improve inactivation efficiency. Addition of glutaraldehyde may also be considered, because it is not only efficient against spores [43] but also against viruses and vegetative bacteria [39]. Low concentration of GA will minimize GA interference with the detection of viruses by negative staining EM [3,20]. However, it cannot be excluded that addition of GA may interfere with the FA inactivation. Further experiments are necessary to clearly prove efficiency of the proposed protocols for bacteria.

In summary, the application of the inactivation protocols as suggested in this paper bears a residual risk of incomplete inactivation, which cannot be quantified so far. Since the negative staining procedure and electron microscopy itself seem not to inactivate virus samples [3], a residual activity of the inactivated samples prepared for diagnostic electron microscopy must be considered. However, a considerable decrease of the infective dose will be achieved by applying the temperature-shift protocol, which reduces the risk of accidental spread of infectious material significantly. As a consequence, even inactivated samples must be treated by proper means of hygiene, e.g., preparation in a biosafety cabinet, safe transport and decontamination of contaminated items. Future experiments should expand testing of the suggested protocols to high amounts of small non-enveloped viruses, which are considered as the most stable virions in disinfection tests [34,37] and other pathogens under various realistic conditions, to further improve the biosafety of diagnostic EM. Thus far, the temperature-shift protocol is the most efficient and validated inactivation procedure that is available for rapid diagnostic EM of viruses.

## Supplementary Files

Supplementary File 1
