# Supplementary material for: Evaluation of Virus Inactivation by Formaldehyde to Enhance Biosafety of Diagnostic Electron Microscopy
Source: Viruses. 2015 Feb 10;7(2):666–79. doi: 10.3390/v7020666 (PMC4353909; doi:10.3390/v7020666)
Supplement: Supplementary File 1 [file viruses-07-00666-s001.pdf]

Supplementary Materials

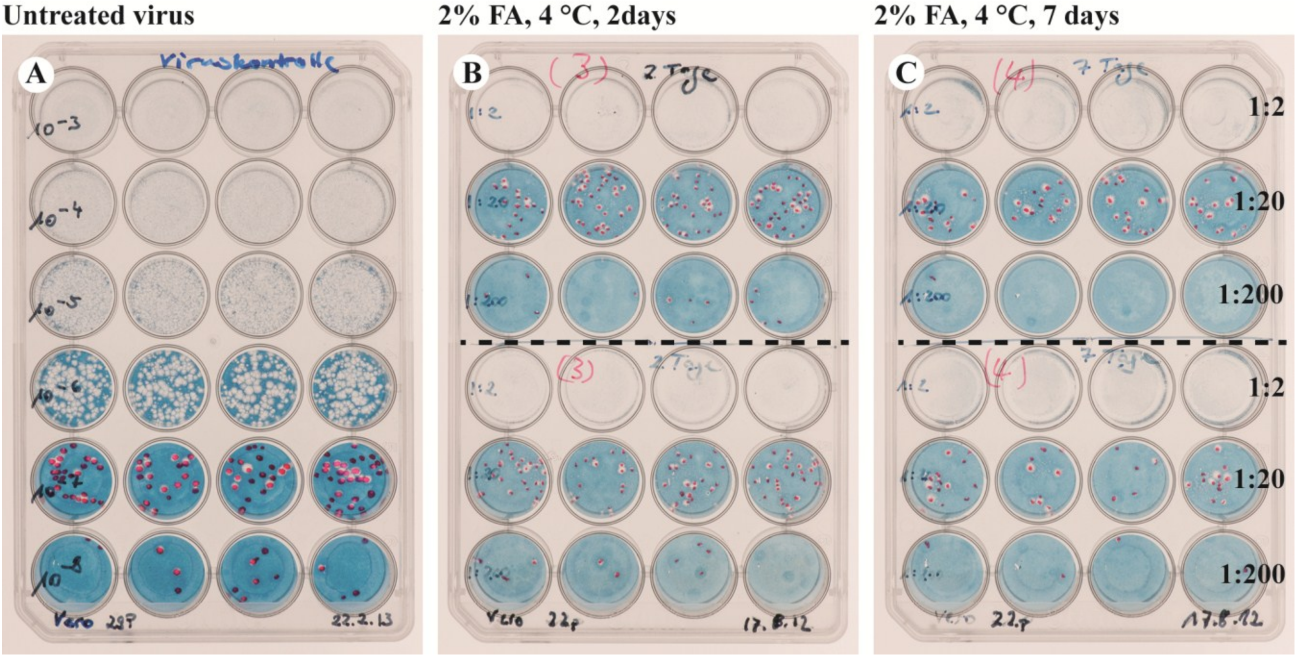

Figure S1. *Cont.*

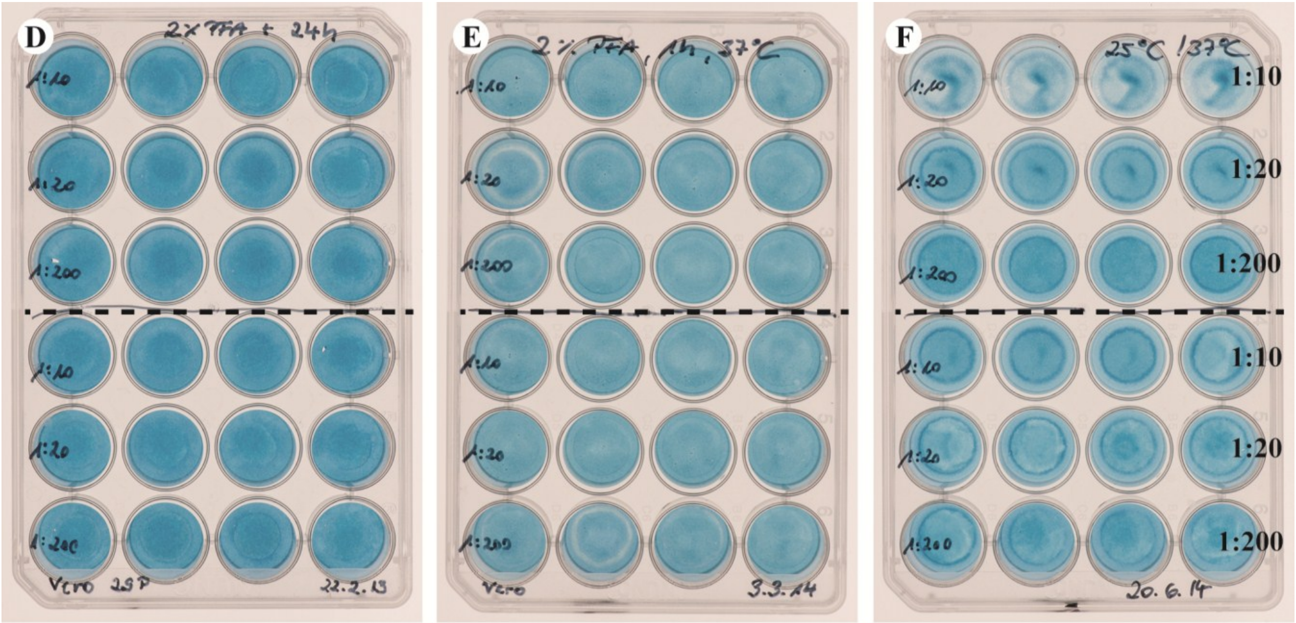

2% FA, 25 °C, 24 hours **2% FA, 37 °C, one hour** **2% FA, 25/37 °C, 30/30 min**

Figure S1. Representative 24-well plates of plaque assays, which were performed to measure infectivity of *Vaccinia virus*. Untreated and treated virus suspensions were added to Vero cells which were seeded on the bottom of the wells. The cells were incubated for four h at 37 °C and then covered with carboxymethyl cellulose. After four days of further incubation, cells were fixed and stained for visualization of infected cell clusters, which appear as bright dots (plaques) among cells stained in blue. Counted plaques are marked with a red dot by using a pen. (**A**) Untreated virus stock suspension (*Vaccinia virus* VR–1536) in tenfold dilution steps (1:10^3 (10^{-3}) to 1:10^8 (10^{-8}); 4 wells per dilution). The first countable dilution was 1:10^7 (10^{-7}). At lower dilution, number of cells infected was too high, resulting in fused plaques or entirely removed cells; (**B**)–(**F**) FA–treated virus suspensions. In all cases, a concentration of 2% FA was used for inactivation. In each plate, three dilutions (1:2 to 1:200 or 1:10 to 1:200; 4 wells per dilution) of the treated suspensions were tested in duplicate (one above and one below the dashed line). Bright dots not marked in red were interpreted as scratches and were not counted; (**B**) Examples for incomplete inactivation after treatment with 2% FA for two days and (**C**) seven days at 4 °C; Blank wells in (**B**) and (**C**) are due to cytotoxicity of the 1:2 dilution. The presence of plaques indicates virus infectivity; (**D**) No plaques are visible after 24 h incubation at 25 °C, (**E**) after one hour at 37°C and (**F**) after half an hour at 25 °C followed by half an hour at 37 °C (temperature–shift protocol) indicating complete virus inactivation.
